# Supplementary material for: The art of valuation: Using visual analysis to price classical paintings by Swedish Masters
Source: PLoS One. 2024 Jan 19;19(1):e0296906. doi: 10.1371/journal.pone.0296906 (PMC10798490; doi:10.1371/journal.pone.0296906)
Supplement: S1 Appendix — (DOCX) [file pone.0296906.s002.docx]

**Appendix A. Theme and paintings**

This appendix shows a selection of the images obtained from the auction houses. They illustrate that the classification of a painting is not always clear.

Figure A1: This painting by Anders Zorn is an oil painting with the title “Sunday Morning” (Swedish: “söndag morgon”). Visual inspection results in classification of the painting in the theme ‘people, whereas the terminological labeled it as ‘unclassified’. The painting was sold at Bukowskis in 2021 for SEK million 35.2 (excl. commission). Photograph from Bukowskis, who provided a CC.BY license.


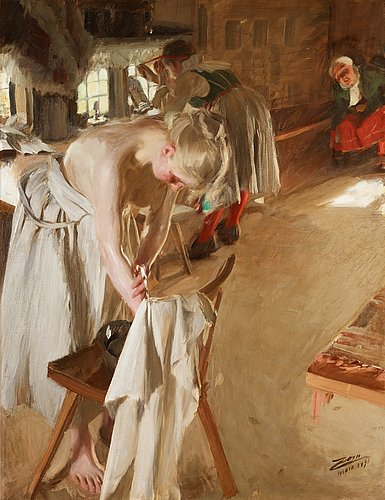


Figure A2: This painting by Jenny Nyström is a water color titled “Christmas Fun” (Swedish: “Julglädje”. Visual inspection results in classifying it in the theme ‘people’, whereas the terminological labeled it as ‘unclassified’ The painting was sold at Uppsala Auktionskammare in 2015 for SEK 52,000 (excl. commission). Photograph from Uppsala Auktionskammare, who provided a CC.BY license.


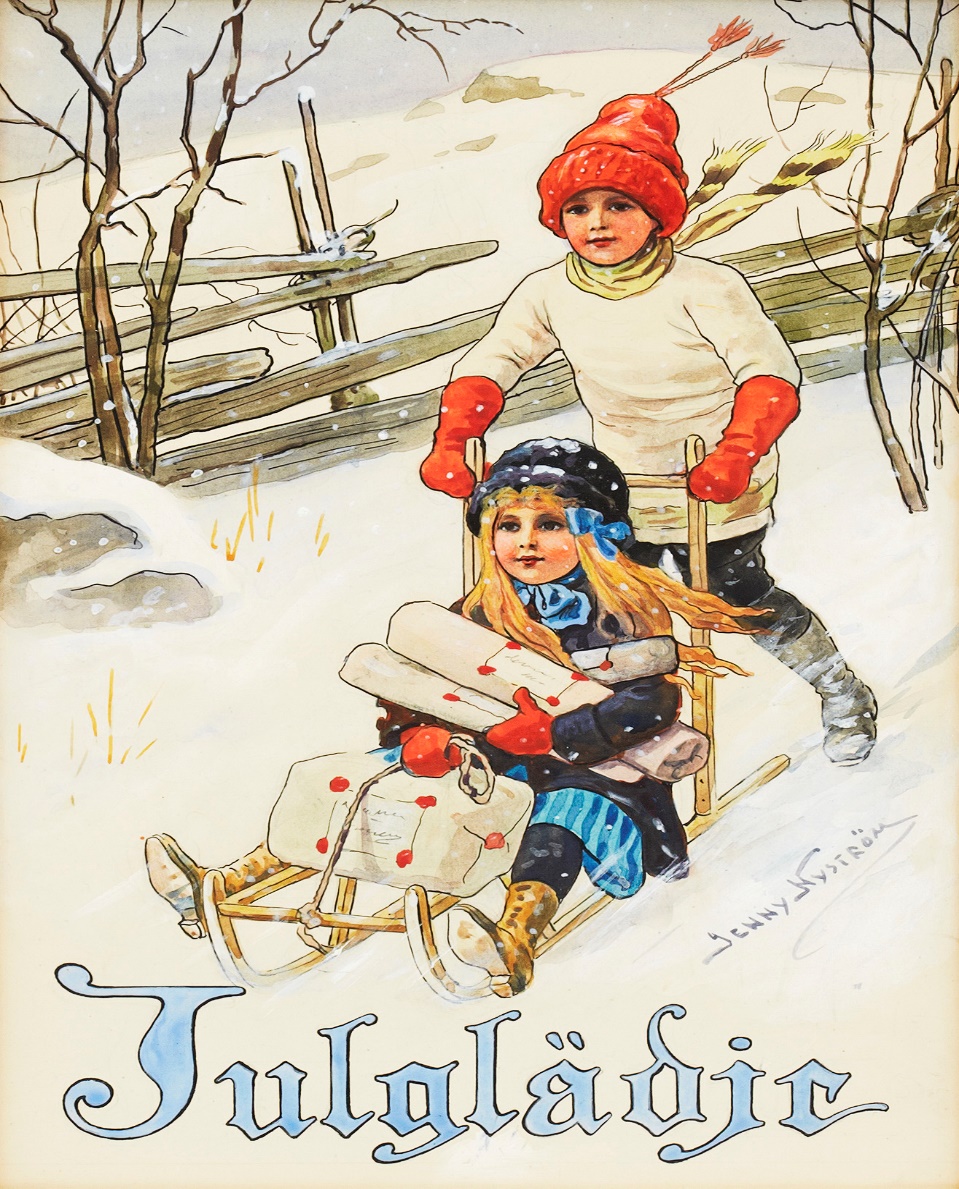


Figure A3: This oil painting by Anders Zorn is “Red Sand” (Swedish: Röd sand). Visual inspection results in classifying it in the theme “Nude”, whereas the terminological labeled it as ‘unclassified’. The painting was sold at Bukowskis in 2010 for SEK million 11.8 (excl. commission). Photograph from Bukowskis, who provided a CC.BY license.


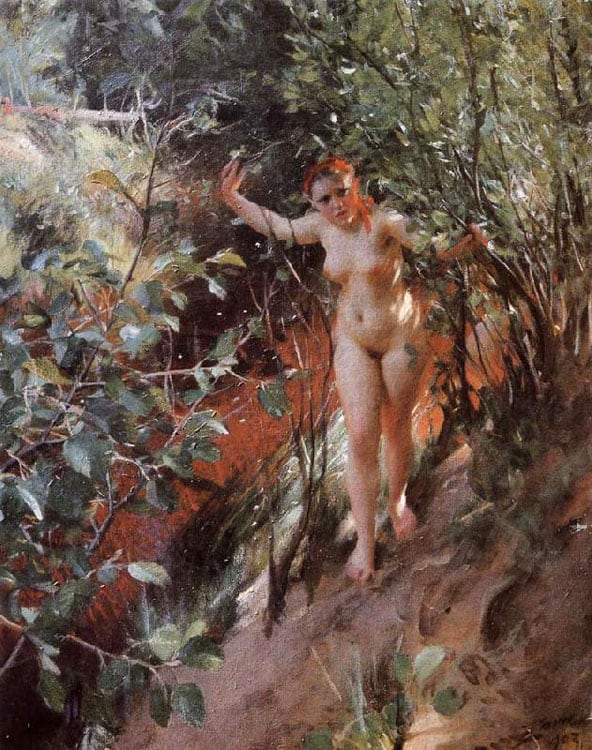


Figure A4: This oil painting by Anders Zorn is “Old Mirror” (Swedish: Gammal spegel). Visual inspection results in classifying it in the theme “Nude”, whereas the terminological labeled it as ‘unclassified’. The painting was sold at Bukowskis in 2020 for SEK million 3.6 (excl. commission). Photograph from Bukowskis, who provided a CC.BY license.


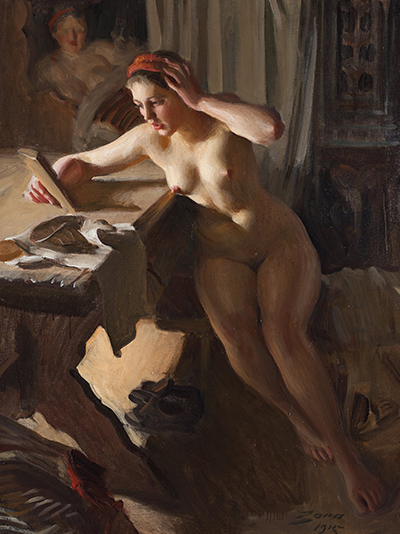


**Appendix B. Distribution of hammer prices and theme.**

| Panel A: Distribution of hammer prices (SEK,000’) | | | | | | | | | | | | | |
| --- | --- | --- | --- | --- | --- | --- | --- | --- | --- | --- | --- | --- | --- |
| Percentiles | (1)  All | | | | (2)  Oil | (3)  Water color | | | | (4)  Pastel | | (5)  Mixed | |
| Lowest | 2.3 | | | | 3.7 | 3.4 | | | | 4.3 | | 2.3 | |
| 1% | 6.5 | | | | 6.8 | 6.5 | | | | 4.3 | | 5.4 | |
| 5 | 9.6 | | | | 10.2 | 9.5 | | | | 8.0 | | 7.3 | |
| 10 | 12.6 | | | | 13.4 | 11.8 | | | | 9.1 | | 9.1 | |
| 25 | 19.4 | | | | 21.2 | 18.0 | | | | 13.4 | | 14.0 | |
| 50 (median) | 36.6 | | | | 39.8 | 34.4 | | | | 21.5 | | 25.5 | |
| 75 | 90.2 | | | | 95.7 | 102.7 | | | | 63.8 | | 45.2 | |
| 90 | 268.8 | | | | 251.8 | 597.8 | | | | 118.3 | | 82.0 | |
| 95 | 600.0 | | | | 511.9 | 1,864.9 | | | | 297.8 | | 145.0 | |
| 99 | 4,340.3 | | | | 4,108.0 | 5,564.5 | | | | 1,075.0 | | 282.0 | |
| Highest | 29,312.4 | | | | 16,213.0 | 29,312.4 | | | | 1,075.0 | | 815.9 | |
| Mean | 215.4 | | | | 197.6 | 370.6 | | | | 66.5 | | 42.1 | |
| Std.dev. | 996.0 | | | | 868.1 | 1,557.5 | | | | 144.2 | | 63.5 | |
| Skewness | 12.31 | | | | 10.07 | 10.71 | | | | 5.18 | | 6.71 | |
| Kurtosis | 228.11 | | | | 125.38 | 161.9 | | | | 33.84 | | 70.75 | |
| Observations (%) | 4,873 | | | | 3,601 (73.90) | 860 (17.65) | | | | 77 (1.58) | | 335 (6.87) | |
| Panel B: Hammer prices (SEK,000’) by theme (visual inspection) | | | | | | | | | | | | | |
|  | (1) | | (2) | | (3) | (4) | | (5) | | (6) | (7) | | (8) |
| Percentiles | Landscape | | Portrait | | Urban | Animal | | Object | | People | Still life | | Nude |
| Lowest | 2.3 | | 3.7 | | 3.2 | 5.2 | | 4.6 | | 5.3 | 5.4 | | 7.9 |
| 1% | 6.5 | | 5.6 | | 6.5 | 7.5 | | 5.6 | | 6.5 | 5.6 | | 7.9 |
| 5 | 9.7 | | 9.0 | | 9.1 | 12.9 | | 9.6 | | 9.0 | 10.8 | | 13.4 |
| 10 | 12.9 | | 11.8 | | 11.3 | 16.1 | | 12.2 | | 10.8 | 15.1 | | 18.8 |
| 25 | 19.4 | | 20.0 | | 17.2 | 27.8 | | 18.4 | | 16.1 | 21.5 | | 30.4 |
| 50 (median) | 35.6 | | 41.3 | | 30.0 | 80.6 | | 34.2 | | 30.1 | 32.3 | | 360.0 |
| 75 | 72.3 | | 133.0 | | 60.0 | 161.3 | | 72.3 | | 61.3 | 53.8 | | 2,196.6 |
| 90 | 193.5 | | 616.2 | | 123.2 | 319.2 | | 161.1 | | 141.0 | 186.2 | | 7,790.3 |
| 95 | 414.0 | | 1,797.3 | | 205.4 | 600.0 | | 340.5 | | 268.8 | 329.8 | | 12,093.8 |
| 99 | 4,108.0 | | 5,375.0 | | 1075.0 | 2,580.0 | | 4,811.6 | | 1,848.6 | 418.4 | | 13,247.0 |
| Highest | 12,631.3 | | 12,241.9 | | 14,891.5 | 9,782.5 | | 29,312.4 | | 8,600.0 | 445.2 | | 13,247.0 |
| Mean | 173.9 | | 319.6 | | 99.7 | 206.1 | | 213.7 | | 121.9 | 64.8 | | 2,101.5 |
| Std,dev, | 813.3 | | 998.9 | | 632.0 | 693.2 | | 1,646.8 | | 581.1 | 88.2 | | 3,522.8 |
| Skewness | 9.84 | | 6.8 | | 20.02 | 10.25 | | 14.76 | | 11.41 | 2.65 | | 2.02 |
| Kurtosis | 116.68 | | 53.30 | | 449.68 | 126.33 | | 241.41 | | 152.01 | 9.42 | | 5.97 |
| Observations (%) | 1,540 (31.60) | | 954 (19.58) | | 685 (14.06) | 665 (13.65) | | 444 (9.11) | | 327 (6.71) | 199 (4.08) | | 59 (1.21) |
| Panel C: Hammer prices (SEK,000’) by theme (terminological approach) | | | | | | | | | | |  | | |
|  | (1) | (2) | | (3) | (4) | (5) | (6) | | (7) | (8) | (9) | | |
| Percentiles | Landscape | Portrait | | Urban | Animal | Object | People | | Still life | Nude | No Theme | | |
| Lowest | 4.2 | 3.7 | | 5.4 | 5.2 | 8.6 | 5.6 | | 5.4 | 7.9 | 2.3 | | |
| 1% | 5.8 | 4.6 | | 6.3 | 7.6 | 8.6 | 6.7 | | 5.6 | 7.9 | 6.5 | | |
| 5 | 9.6 | 6.7 | | 9.4 | 16.0 | 11.1 | 8.9 | | 9.7 | 9.9 | 9.6 | | |
| 10 | 12.4 | 9.4 | | 11.6 | 20.2 | 12.2 | 11.1 | | 14.0 | 13.9 | 12.8 | | |
| 25 | 18.3 | 17.1 | | 17.2 | 39.5 | 17.2 | 16.4 | | 21.5 | 20.8 | 19.4 | | |
| 50 (median) | 33.5 | 36.6 | | 27.9 | 101.4 | 29.5 | 30.4 | | 31.7 | 40.8 | 37.0 | | |
| 75 | 75.5 | 90.9 | | 59.1 | 164.3 | 516 | 86.0 | | 51.6 | 102.9 | 89.0 | | |
| 90 | 188.3 | 209.6 | | 129.0 | 288.9 | 87.2 | 270.6 | | 120.8 | 205.5 | 340.5 | | |
| 95 | 344.0 | 296.7 | | 250.0 | 560.0 | 376.3 | 543.9 | | 215.0 | 567.5 | 913.8 | | |
| 99 | 1,773.8 | 600.0 | | 3,004.8 | 5,840.6 | 410.8 | 3,100.0 | | 355.6 | 875.0 | 5375.0 | | |
| Highest | 12,631.3 | 645.0 | | 9,695.6 | 9,782.5 | 410.8 | 10,200.0 | | 376.3 | 875.0 | 29,312.4 | | |
| Mean | 122.8 | 76.8 | | 130.1 | 260.2 | 53.5 | 194.8 | | 53.6 | 107.1 | 264.8 | | |
| Std,dev, | 578.2 | 106.9 | | 696.6 | 949.6 | 83.0 | 849.9 | | 66.3 | 191.4 | 1,170.5 | | |
| Skewness | 15.50 | 3.01 | | 485,215.9 | 7.96 | 6,895.4 | 9.59 | | 3.01 | 3.47 | 11.20 | | |
| Kurtosis | 294.43 | 13.70 | | 139.9 | 71.56 | 14.87 | 107.97 | | 12.43 | 14.41 | 187.9 | | |
| Observations (%) | 847 (17.38) | 120 (2.46) | | 290 (5.95) | 330 (6.77) | 58 (1.19) | 183 (3.76) | | 138 (2.83) | 20 (0.41) | 2,887 (59.24) | | |

Notes: This table shows in Panel A the distribution and summary statistics of hammer prices for all paintings and technique used. In Panel B and C, we report results for all paintings together with the theme: landscape, portrait, urban, animal, object, people, still life and nude. All paintings in Panel B have been classified by visualization of each painting. All paintings in Panel C have been classified by a terminological approach as in Renneboog and Spaenjers (2013). Hammer prices are expressed in 2020 price level and in Swedish Krona (SEK) where we use the consumer price index from Statistics Sweden as a deflator, Data reflects paintings sold at three auction houses over the period 2010 to 2020.

**Appendix C. Art paintings used in the robustness test of theme classification**

This appendix shows the ten paintings used in the robustness tests for thematic classification (landscape, portrait, object, urban (street, markets, harbor), people (family), still life, animal and nude).

Figure A5: This painting by Julia Beck is an oil painting with the title “Landscape in haze” (Swedish: “Landskap i dis”). Visual inspection results in classification of the painting in the theme ‘landscape’. The terminological labeled it as ‘landscape’. The painting was sold at 2010 in Bukowskis for SEK 21,421 (excl. commission). Photograph from Bukowskis, who provided a CC.BY license.


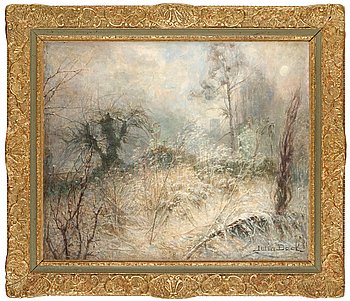


Figure A6: This painting by Carl Fredrik Hill is an oil painting with the title “Landscape” (Swedish: “Landskap”). Visual inspection results in classification of the painting in the theme ‘landscape’. The terminological labeled it as ‘landscape’. The painting was sold at Bukowskis in 2010 for SEK 326,946 (excl. commission). Photograph from Bukowskis, who provided a CC.BY license.


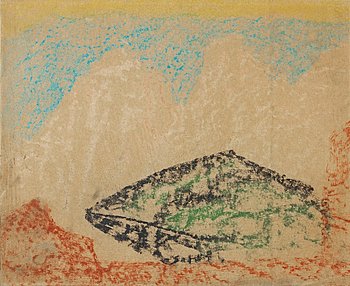


Figure A7: This painting by Carl Wilhemson is an oil painting with the title “The old ladies” (Swedish: “Gummorna”). Visual inspection results in classification of the painting in the theme ‘portrait’, whereas the terminological labeled it as ‘unclassified’. The painting was sold at Bukowskis in 2019 for SEK 166,155 (excl. commission). Photograph from Bukowskis, who provided a CC.BY license.


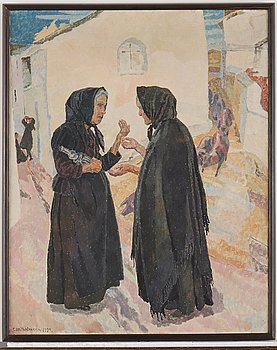


Figure A8: This painting by John Bauer is a water color painting with the title “King Helamund and the castle council” (Swedish: “Kung Helamund och slottsrået”). Visual inspection results in classification of the painting in the theme ‘portrait’, whereas the terminological labeled it as ‘unclassified’. The painting was sold at Bukowskis in 2019 for SEK 213,610 (excl. commission). Photograph from Bukowskis, who provided a CC.BY license.


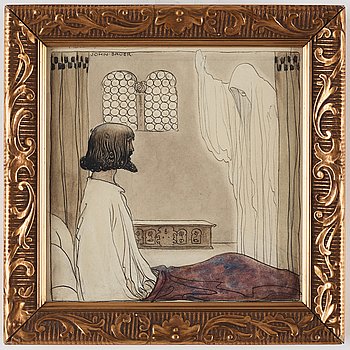


Figure A9: This painting by Anshelm Scultzberg is an oil painting with the title “Winter landscape, motif from the area between Mora and Älvdalen” (Swedish: “Vinterlandskap, motiv från trakten mellan Mora och Älvdalen”). Visual inspection results in classification of the painting in the theme ‘landscape’. The terminological labeled it as ‘landscape’. The painting was sold at Auktionsverket in 2010 for SEK 19,166 (excl. commission). Photograph from Auktionsverket, who provided a CC.BY license.


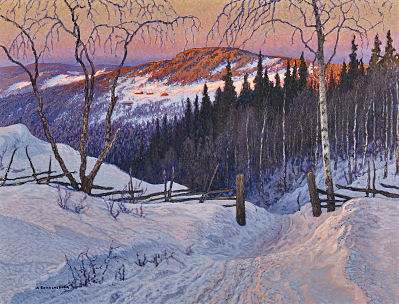


# Figure A10: This painting by Bruno Liljefors is an oil painting with the title “Ducks and Ducklings in a Creek

” (Swedish: “Andfamilj i vårvassen”). Visual inspection results in classification of the painting in the theme ‘animal’. The terminological labeled it as ‘animal’. The painting was sold at Auktionsverket in 2013 for SEK 213,000 (excl. commission). Photograph from Auktionsverket, who provided a CC.BY license.

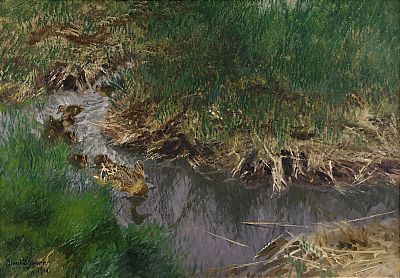


Figure A11: This painting by Per Ekström is an oil painting with the title “Sunset over Alvaret” (Swedish: “[Solnedgång över Alvaret](https://www.mutualart.com/Artwork/Solnedgang-over-Alvaret/AC5666AE6364F9A5)”). Visual inspection results in classification of the painting in the theme ‘landscape’, whereas the terminological labeled it as ‘unclassified’. The painting was sold at Bukowskis in 2020 for SEK 36,000 (excl. commission). Photograph from Bukowskis, who provided a CC.BY license.
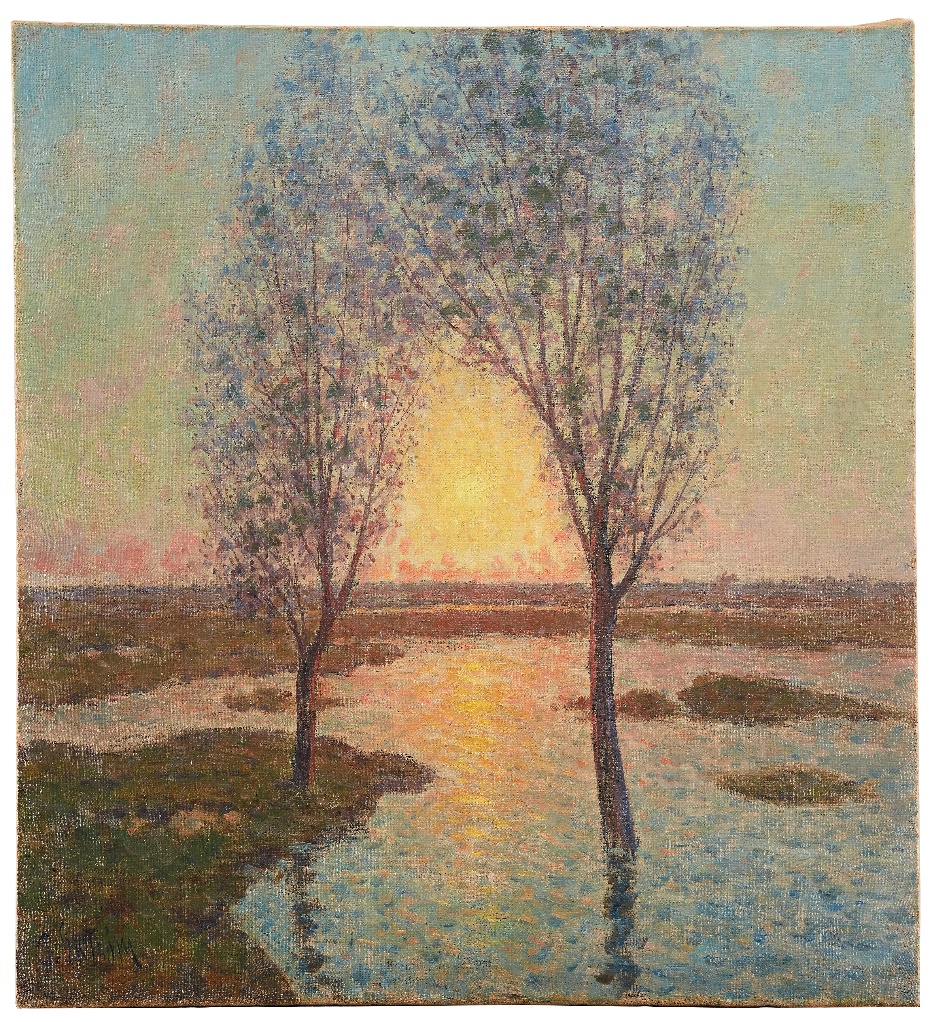


Figure A12: This painting by Helmer Osslund is an oil painting with the title “The Indal river” (Swedish: “Indalsälven”). Visual inspection results in classification of the painting in the theme ‘landscape’, whereas the terminological labeled it as ‘unclassified’. The painting was sold at Uppsala Auktionskammare in 2016 for SEK 266,000 (excl. commission). Photograph from Uppsala Auktionskammare, who provided a CC.BY license.


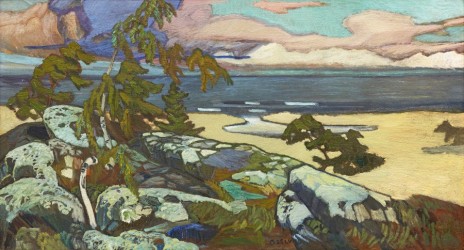


Figure A13: This painting by Helmer Osslund is an oil painting with the title “Autumn” (Swedish: “Höst”). Visual inspection results in classification of the painting in the theme ‘landscape’. The terminological labeled it as ‘landscape’. The painting was sold at Auktionsverket in 2013 for SEK 27.950 (excl. commission). Photograph from Auktionsverket, who provided a CC.BY license.


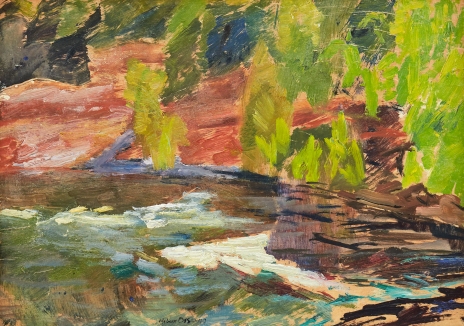


Figure A14: This painting by Severin Nilsson is an oil painting with the title “Playing child with Swedish flag” (Swedish: “Lekande barn med svenska flaggan”). Visual inspection results in classification of the painting in the theme ‘people’, whereas the terminological labeled it as ‘unclassified’. The painting was sold at Auktionsverket in 2010 for SEK 20,293 (excl. commission). Photograph from Auktionsverket, who provided a CC.BY license.


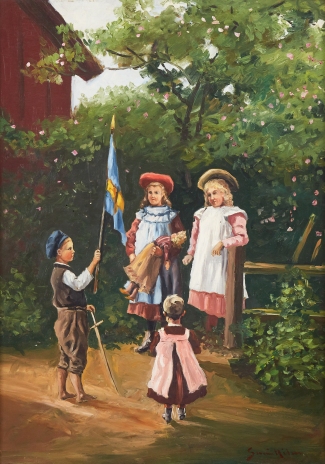


**Appendix D: Distribution of paintings across theme**

| Thematic approach | Landscape | Portrait | Urban | Animal | Object | People | Still life | Nude | No theme |
| --- | --- | --- | --- | --- | --- | --- | --- | --- | --- |
| Visual: |  |  |  |  |  |  |  |  |  |
| N | 1,984 | 1,386 | 929 | 872 | 587 | 460 | 270 | 78 | n/a |
| (%) | (30.22) | (21.11) | (14.15) | (13.28) | (8.94) | (7.01) | (4.11) | (1.19) |  |
| Terminological: |  |  |  |  |  |  |  |  |  |
| N | 1,122 | 163 | 397 | 434 | 79 | 260 | 191 | 27 | 3,893 |
| (%) | (17.09) | (2.48) | (6.05) | (6.62) | (1.20) | (3.96) | (2.91) | (0.41) | (59.29) |

Notes: The data reflect a total of 6,566 paintings from art auctions from three Swedish auction houses over the time period 2000 to 2020.

**Appendix E: Matching coefficients between the visual and terminological approach**

|  |  | Terminological approach | | | | | | | |
| --- | --- | --- | --- | --- | --- | --- | --- | --- | --- |
|  |  | Landscape | Portrait | Urban | Animal | Object | People | Still life | Nude |
| Visual approach | Landscape | 0.8041 |  |  |  |  |  |  |  |
|  | Portrait |  | 0.8113 |  |  |  |  |  |  |
|  | Urban |  |  | 0.8852 |  |  |  |  |  |
|  | Animal |  |  |  | 0.9217 |  |  |  |  |
|  | Object |  |  |  |  | 0.9108 |  |  |  |
|  | People |  |  |  |  |  | 0.9007 |  |  |
|  | Still life |  |  |  |  |  |  | 0.9864 |  |
|  | Nude |  |  |  |  |  |  |  | 0.9895 |

Notes: The themes along the rows are those assigned via visual inspection. The themes along the columns are those assigned via the terminological approach. The coefficient shows the match percent. That is, the ratio of total matches to the total population. Please note than when themes are assigned via the terminological approach, they could also be assigned to ’No theme’ if the approach is unsuccessful in assigning the painting into one of the other themes. Since no painting were allocated to ’No theme’ via the visual approach, the matching percentage is 0.

**Appendix F: Systematic bias check**

|  | (1) |
| --- | --- |
| Variable | Not assigned |
| *Artist characteristics:* |  |
| Unsigned (Yes=1) | -0.021 |
|  | (0.030) |
| *Painting characteristics:* |  |
| *Size of painting* |  |
| Surface (cm^2^) | 0.000** |
|  | (0.000) |
| Surface squared | -0.000 |
|  | (0.000) |
| *Technique (Base type: Oil)* |  |
| Mixed | 0.044 |
|  | (0.029) |
| Pastel | 0.028 |
|  | (0.044) |
| Water color | 0.078*** |
|  | (0.025) |
| *Theme (Base theme: Landscape):* |  |
| Animal | 0.056* |
|  | (0.033) |
| Nude | 0.148** |
|  | (0.058) |
| Object | 0.277*** |
|  | (0.023) |
| People | 0.223*** |
|  | (0.026) |
| Portrait | 0.096*** |
|  | (0.023) |
| Still life | -0.179*** |
|  | (0.055) |
| Urban | 0.140*** |
|  | (0.022) |
| *Sales characteristics:* |  |
| *Auction house (base Auktionsverket)* |  |
|  |  |
| Bukowskis | 0.060*** |
|  | (0.014) |
| Uppsala Auktionskammare | 0.007 |
|  | (0.016) |
| *Winter sale dummy:* |  |
| December | 0.010 |
|  | (0.012) |
| Year fixed effects | Yes |
|  |  |
| Artist fixed effects | Yes |
|  |  |
| Constant | 0.764*** |
|  | (0.061) |
| Observations | 6,566 |

Notes: The results in column (1) is from a Linear Probability Model where the dependent variable, ‘Not Assigned’ is equal to 1 if the painting did not get assigned a theme based on the terminological approach of Renneboog and Spaenjers (2013), and 0 otherwise. Artist fixed effects and Year dummies are included in estimation of the regression models, but not reported. Due to the large number of artists in our sample, artist with less than 10 paintings in our sample are grouped together as “Other artists”.
