# Supplementary material for: The art of valuation: Using visual analysis to price classical paintings by Swedish Masters
Source: PLoS One. 2024 Jan 19;19(1):e0296906. doi: 10.1371/journal.pone.0296906 (PMC10798490; doi:10.1371/journal.pone.0296906)
Supplement: Attachment — Submitted filename: PONE-D-23-12660.R1 - Response to reviewers.docx [file pone.0296906.s004.docx]

PONE-D-23-12660-R.1

The Art of Valuation:

Using Visual Analysis to Price Classical Paintings by Swedish Masters

Dear Editor.

Thank you very much for your invitation to revise and resubmit our paper. We appreciate the reviewers’ comment and suggestions and did our utmost best to comply and to integrate in the revision. A detailed response goes below.

We hope you and the reviewers find the points raised adequately addressed and we look forward to your response.

Yours sincerely,

==================================================================

Reviewer #1:

The authors addressed all the comments from the previous review and introduced a comparison between terminological and visual inspection of the artworks, resulting in interesting findings. In what follows, I report a series of points that I think the authors should consider:

1) The authors introduced an artist fixed effect, which allowed them to align their results with previous studies, since a name effect is actually important in shaping the prices. However, the authors discuss the different prices obtained by male artists' pieces with respect to female artists' ones, using a dummy in their estimations. This is strange, since the artist fixed effect should capture all the artist-specific characteristics (among which gender), so every artist-specific variable could not actually be estimated if an artist fixed effect is introduced. The reason why the gender dummy has actually a coefficient notwithstanding is only reported in the footnote of the Tables 3 and 4 and the Table in appendix F, namely the authors pooled together the artists with less than 10 pieces, however this suggests that the gender dummy and the artist fixed effects are likely to be highly correlated (there is no perfect multicollinearity since some artists are pooled together, but there would be perfect multicollinearity if one would run the regression without the pooled artists). This likely inflates the standard errors, and the issue is likely to be worse as the number of pooled artists is lower. I believe that omitting the gender dummy would be the most effective means to solve this issue.

*We first want to thank the reviewer for thoroughly studying our paper again and providing such thoughtful and helpful comments. As to the issue of the fixed effects in relation to artist gender, we find the reviewer is absolutely right. The inclusion of a gender dummy is of little meaning in the current setting with artist fixed effects. The dummy for gender has therefore been removed from the analysis, as the effect is rightly captured by the inclusion of artist fixed effects. Furthermore, we want to point out that pooling the artists with less than 10 paintings together is needed given the structure of the data. In our data from the three auction houses, we have a large number of artists, many of whom have sold a very limited number of paintings. Often this is only one painting, or if more than one, then they were all sold at the same auction. We therefore have a setting with many small, and unbalanced group sizes. We are therefore unable to identify any within group variation for such artist (an unidentifiability problem). Furthermore, in a setting with a limited dependent variable model estimated via maximum likelihood (such as the first stage in a Heckman selection model), including fixed effects when group sizes are small will substantially bias the estimates away from zero, with the bias diminishing with increasing group size (see also Greene, 2004).*

*One solution to this issue is to estimate a random effects Heckman selection model. However, despite the potential increased efficiency of such estimator, it seems very unreasonable in this setting to assume no correlation between the artist fixed effects, and the remaining right-hand side variable. Thus, such an approach would lead to biased and inconsistent estimates. Another solution is to combine the small groups, which is the solution we opted for here. Many other authors are not faced with this issue, as their data has fewer number of artist and/or more observations per artist, with sales spread out over multiple time periods, or they only differentiate if a painting has a name/signature or not. Examples are Angelini et al (2023), Hernando & Campo (2017), Ma et al. (2022), Radermecker & Anne-Sophie (2019), Renneboog & Spaenjers (2013).*

2) I appreciate the effort the authors made in carrying on the terminological analysis and the comparison with the visual inspection analysis. I think it should be made clearer, though, which was the language used. In particular, were all the artworks' title in Swedish? If so, did the authors traduce the titles in English and/or French to use the Renneboog & Spaenjers (2013) methodology (namely, the keywords they report in their Appendix B)? Or has the methodology been applied after translating the keywords for each theme in Swedish? This information is needed to allow for the replicability of the study. I think the authors may also want to replicate what Renneboog and Spanjers did in their Appendix B, reporting which words have been used for each theme.

*In our classification of paintings into topics, we use the titles as reported in the auction catalogues. With a few exceptions, all titles are in Swedish. An example of a painting in another language is when the painting represents a person, for example the painting “Mrs Emily Crane” by Anders Zorn. The classification of each painting is carried out after translating the titles and keywords from Renneboog and Spaenjers (please see their Appendix B) into Swedish. Although we follow their methodology, we use the full name of each painting. We believe this approach is appropriate as several paintings titles begins with “On the way”, “Art to” etc. We did not detect, following visual inspection, paintings which could be attributed to ABSTRACT, RELIGION, or UNTITLED (due to the sample paintings). However, we added a new category, OBJECT, as it illustrates a “wall”, “sailboat”, “boat”, “a lighthouse”, “castle”, “beehive”, “apple”, “orange”, “grape”, “rain” or “cloud”. The classification into topic categories was executed using Swedish.*

*The categories and the search strings used in our paper is as follows:*

*ANIMAL	(horse, bird, elk, fox, dog, cats, sheep, hen, cock, geese, goose)*

*LANDSCAPE (landscape, country landscape, coastal landscape, seascape, winter landscape, snow landscape, mountain, lake, sea, cliffs, valley)*

*NUDE	(model, nude)*

*OBJECT	(wall, sailboat, boat, lighthouse, castle, wall, beehive, apple, orange, grape, rain, cloud)*

*PEOPLE	(people, person, mr, mrs, herr, fru, family, boy, girl, man, woman, child, infant, kid, baby)*

*PORTRAIT	(portrait, self-portrait)*

*STILL LIFE	(still life, nature, flower, flower wase, bouquet)*

*URBAN	(city, town, village, street, harbor, port, Stockholm, Visby, Öland, Arild, Bretagne, London, Paris, Västkusten)*

Some minor points:

1) The authors state that the standard approach in the analysis of artwork prices has been to focus on sold paintings only (page 6, line 71 and page 7, line 100). In the previous round of review I listed some exception, as an example, but after the paper of Collins et al. (2009), a lot of articles started taking into account the existence of a possible selection bias (Google Scholar lists 87 citations of Collins et al. 2009, as for today). So it might not be appropriate to state that focusing on sold artworks only is the standard approach, since several works in the last years take selection bias into account.

*We have reformulated our statements.*

2) I think uniforming the terminology throughout the paper would help the reader: sometimes the terminilogical approach is called semantic method or semantic approach; similarly, the art-informed inspection is referred to as visual inspection (or as "artistic information" in Appendix C, which I think is even misleading).

*The naming convention has been cleaned up and made consistent throughout the paper; we label as terminological approach throughout and refrain from suggesting that the method relies on artistic information.*

3) On page 13-14, lines 257-261, the authors refer to visual inspection themes, reporting the percentage of each theme. However, the visual inspection themes are introduced only later on (page 15). It would maybe be better to report these percentages after the introduction of the themes.

*We have moved this reference to Appendix D, so it is now better aligned with the rest of the paper on we expect it will not lead to any confusion.*

4) Appendix E contains a table which reports the matching labels using the two methods. The comparison is made using only the subsample for which the terminological approach attaches a label different from "N/A" to the artworks. I think that it might be more informative to have the table reporting the matching labels for all the sample, since the coefficients currently reported in the table may suggest that the matching is not that bad. This would also better highlight the interesting result the authors find, about measurement error (page 23, line 476-478).

*Appendix E has been updated to reflect this suggestion.*

5) In the paper, lambda is incorrectly spelled as "lamba" (page 23, lines 479 and 481).

*Fixed this*

6) On page 20, line 418, "tech (pastel)" should read "technique (pastel)".

*Fixed this*

7) On page 21, line 443-444, the authors write "unsigned works deliver on average a 34% discount compared to unsigned work." It should be "compared to signed works".

*Fixed this*

Reviewer #2:

Though you provided three additional citations to discuss the state-of-the art techniques in extracting painting themes, I don't feel the current response is adequate in elaborating these recent progresses. I also noticed that R1 raised a similar point that "Recent works (Tan et al. 2016; Sheppard 2021; Choi et al. 2023) also implement what the authors suggest in their future extensions (or something along these lines), namely “use artificial intelligence” (p. 21, line 495)." And you simply cited R1's suggested references without clear elaboration. I would recommend spend a few paragraphs in the front part of your manuscript to introduce these progress, discuss them in detail, compare those works with your project. This will help readers better position the contributions of this work compared to traditional and most cutting edge techniques.

*Thank you for your suggestion. We elaborate on these developments in the revised paper’s introduction. However, we are sure you are well aware of the fact that we have to keep the study short and don’t want to suggest we envision this study to be a complete review of the literature as ours in a research paper. We refrain from referring to using AI for visual inspection. It is hard to include a comparison between AI approaches and our visual inspection as we are not aware of these techniques being applied to our sample paintings. We believe our relative advantage is in machine learning regarding artwork properties and have started to work on this already.*

*References:*

*Angelini, F., Castellani, M., & Pattitoni, P. (2023). Artist Names as Human Brands: Brand Determinants, Creation and co-Creation Mechanisms. Empirical Studies of the Arts, 41(1): 80–107.* [*https://doi.org/10.1177/02762374211072964*](https://doi.org/10.1177/02762374211072964)

*Greene, W. (2004). The behaviour of the maximum likelihood estimator of limited dependent variable models in the presence of fixed effects. The Econometrics Journal, 7(1): 98-119.*

*Hernando, E., & Campo, S. (2017). Does the Artist’s Name Influence the Perceived Value of an Art Work? International Journal of Arts Management, 19(2): 46–58.*

*Ma, M.X., Noussair, C.N., Renneboog, L. (2022). Colors, Emotions, and the Auction Value of Paintings, European Economic Review, 142, https://doi.org/10.1016/j.euroecorev.2021.104004.*

*Radermecker, A.V.E. (2019). Artworks without names. Journal of Cultural Economics, 43 (3): 443-483.* [*https://doi.org/10.1007/s10824-019-09344-5*](https://doi.org/10.1007/s10824-019-09344-5)

*Renneboog, L. and Spaenjers, C. (2013) ‘Buying Beauty: On Prices and Returns in the Art Market’, Management Science, 59:36–53.*
